# Health Care Professional Association Agency in Preparing for Artificial Intelligence: Protocol for a Multi-Case Study

**DOI:** 10.2196/27340

**Published:** 2021-05-19

**Authors:** Caitlin Gillan, Brian Hodges, David Wiljer, Mark Dobrow

**Affiliations:** 1 Institute of Health Policy, Management, and Evaluation University of Toronto Toronto, ON Canada; 2 Joint Department of Medical Imaging Sinai Health/University Health Network/Women's College Hospital Toronto, ON Canada; 3 Department of Radiation Oncology University of Toronto Toronto, ON Canada; 4 University Health Network Toronto, ON Canada; 5 Department of Psychiatry University of Toronto Toronto, ON Canada

**Keywords:** artificial intelligence, health professions, normalization process theory, case study

## Abstract

**Background:**

The emergence of artificial intelligence (AI) in health care has impacted health care systems, including employment, training, education, and professional regulation. It is incumbent on health professional associations to assist their membership in defining and preparing for AI-related change. Health professional associations, or the national groups convened to represent the interests of the members of a profession, play a unique role in establishing the sociocultural, normative, and regulative elements of health care professions.

**Objective:**

The aim of this paper is to present a protocol for a proposed study of how, when faced with AI as a disruptive technology, health professional associations engage in sensemaking and legitimization of change to support their membership in preparing for future practice.

**Methods:**

An exploratory multi-case study approach will be used. This study will be informed by the normalization process theory (NPT), which suggests behavioral constructs required for complex change, providing a novel lens through which to consider the agency of macrolevel actors in practice change. A total of 4 health professional associations will be studied, each representing an instrumental case and related fields selected for their early consideration of AI technologies. Data collection will consist of key informant interviews, observation of relevant meetings, and document review. Individual and collective sensemaking activities and action toward change will be identified using stakeholder network mapping. A hybrid inductive and deductive model will be used for a concurrent thematic analysis, mapping emergent themes against the NPT framework to assess fit and identify areas of discordance.

**Results:**

As of January 2021, we have conducted 17 interviews, with representation across the 4 health professional associations. Of these 17 interviews, 15 (88%) have been transcribed. Document review is underway and complete for one health professional association and nearly complete for another. Observation opportunities have been challenged by competing priorities during COVID-19 and may require revisiting. A linear cross-case analytic approach will be taken to present the data, highlighting both guidance for the implementation of AI and implications for the application of NPT at the macro level. The ability to inform consideration of AI will depend on the degree to which the engaged health professional associations have considered this topic at the time of the study and, hence, what priority it has been assigned within the health professional association and what actions have been taken to consider or prepare for it. The fact that this may differ between health professional associations and practice environments will require consideration throughout the analysis.

**Conclusions:**

Ultimately, this protocol outlines a case study approach to understand how, when faced with AI as a disruptive technology, health professional associations engage in sensemaking and legitimization of change to support their membership in preparing for future practice.

**International Registered Report Identifier (IRRID):**

DERR1-10.2196/27340

## Introduction

### Background

Many health care professionals are on the cusp of welcoming a new partner into their collaborative practice model, artificial intelligence (AI). Recently, there has been significant discussion in lay media about the impact of AI’s integration on the workforce. Advancements in AI in health care in the coming years will require significant attention to employment, training, education, and regulation [1]. As AI strategies are increasingly being developed in health care, it is important to assess how health care professions and their representative associations will be affected by changes to traditional roles and tasks, some of which will be modified or replaced by technological solutions.

### AI in Health Care

Russell and Norvig [2] define AI as “the study of agents that receive percepts from the environment and perform actions.” In its broadest sense, this can be conceived of as a computer system performing some manipulation of an input (ie, informational or sensory data) to create a novel output without active human intervention [3].

AI encompasses a diverse array of technologies, including automation, aspects of robotics, machine learning, and other approaches for interpreting big data. In health care, such innovations are increasingly mastering menial and rule-based tasks or acting as powerful decision-making tools or monitoring systems to support clinicians. Big data, including both structured data, such as demographics and laboratory medicine results, and unstructured data, such as those extracted from electronic clinical notes using natural language processing, have also been used to develop automated clinical decision-making tools and outcome prediction via predictive analytics [4].

Among other uses, AI has been applied in dermatology, radiology, drug reconciliation, and adverse event management, often outperforming internists in extracting relevant information from unstructured free-text or imaging data [4-6], and in optimizing aspects of pharmacy in drug discovery, tracking, and dispensing [7].

AI constitutes a disruptive technology for health care systems, given the spectrum of applications of related technologies and the associated implications for those who interact with it, from practical clinical workflows to the higher-level considerations related to regulation, scopes of practice, and education.

### Potential Professional Impact

From the perspective of human actors in AI-related health care disruption, there are numerous stakeholder groups, from clinical organizations to academic institutions and from professional and regulatory bodies to related industries. AI is a broad phenomenon, not one that is borne and leveraged within a given professional or academic setting, and the expertise in predicting and planning for its impact may not reside in an easily identified individual role or institution. It is not a single strategy whose impact can be quantified, itemized, and considered. Different stakeholders in the *AI in health care* arena may hold different perspectives on the nature, scope, and role of AI in clinical practice. However, although it is important to consider the role of all stakeholders and the interplay and tensions between these roles and interests in future practice, the health professional association has a unique role in representing affected health professions and takes on frontline responsibility for helping health professions navigate changes due to AI. This will be the focus of this study.

### Role of Health Professional Associations

Although there is some important variation in the structure and mandate of the health professional association, its core business is the protection of a defined scope of practice, with the intention of maintaining the exclusivity of practice and protecting its members from encroachment of other groups. health professional associations, thus, serve to bring members of a certain profession into a formal membership structure to self-manage the definition and maintenance of these attributes. To this end, many assume the role of a certifying body, establishing the entry-to-practice standards of the profession, accrediting those academic programs tasked with training to those standards, and serving as a gatekeeper to the profession by granting membership based on meeting those standards. To further maintain exclusivity, health professional associations assume advocacy roles, furthering the image of the profession and often lobbying for protective practice rights through related health legislation.

Health professional associations are well situated to guide and inform service delivery–level change from a system vantage point. It is incumbent on professional bodies to assist their membership in preparing for change and informing the scope of that change. By design, health professional associations have a unique role in establishing and maintaining the sociocultural, normative, and regulative elements of professional scope and practice [8]. These tasks can serve to protect the exclusivity of their membership, restricting access to an established knowledge base to define their role in society [9]. In times of change, they can be pivotal in legitimizing novel practice both to their membership and to other professions and related stakeholders [10]. They often serve to direct other related entities, such as accrediting bodies and academic programs, in defining the evolution of professional scopes of practice and knowledge bases, within the bounds of any existing regulation.

AI heralds a change in health care that will impact many professions. Contrary to much of the lay media hype, most academic reflections on AI as a disruptive technology have posited that professions are not fundamentally at risk of obsolescence with AI, but then suggest that this position is balanced precariously on the condition of health care professionals’ engagement in informing practice evolution [11-14]. As health professional associations begin to consider implications for their practice, some are convening task groups and putting out calls to action to highlight the role that should be taken by the professions to define their own future within an AI-enabled practice environment [15,16]. Recognizing that many manual, repetitive, and primarily rule-based tasks are expected to be outsourced to AI, professions are seeing the need to reposition themselves within the system on multiple levels. Depending on the magnitude of potential change anticipated by an individual health professional association, these might include advocacy for responsible integration of AI, consideration of changing roles within an evolving health care team, and preparedness for interacting directly with novel technologies and data.

### Objectives

In the context of the traditional role of health professional associations as gatekeepers to a protected knowledge base, there is value in exploring how health professional associations engage in sensemaking and relational work to guide and support their membership when faced with disruptive technology. Although AI implementation may be at its nascent stages in most areas of health care, there is also value in beginning to look at the enactment and monitoring of change as well—the form the change is expected to take and how groups go about implementation. This considers the scope and impact of AI as conceived by the health professional association, regardless of formal conventions.

This proposed work will seek to investigate the following research question: When faced with AI as a disruptive technology, how do health professional associations engage in sensemaking and legitimization of change to support their membership in preparing for future practice?

Within the scope of this project, we also hope to consider from whom, and in what ways, health professional associations seek insight to make sense of AI strategies as they relate to their members’ practice; how that contributes to conceptualizing a relational model for interprofessional collaboration in practice; and, finally, to what extent health professional associations engage in reflexive monitoring and feedback to support their memberships through AI-related change.

## Methods

### Overview

The proposed study will take an exploratory multi-case study approach to address these objectives, using the normalization process theory (NPT) as a theoretical context. Approval for this study has been obtained from the University of Toronto Research Ethics Boards (protocol #00038733).

### Theoretical Approach: NPT

Negotiation of new knowledge, roles, and workflows, in light of a technology that will infiltrate significantly into traditional practice boundaries, often requires a process of validating change against a profession’s established (and socially constructed) value and belief systems [9]. An understanding of where national health professional associations envision and position themselves as stakeholders in AI innovation and how they support a membership faced with AI integration can help to inform active strategies for engaging it in preparing for relevant complex interventions and the future of practice.

Complex interventions in health care, such as the integration of a disruptive technology, are those changes to delivery of care that involve several interacting elements that must all be coordinated for the intervention to be successfully and sustainably implemented [17]. The NPT provides a valuable framework to distill the behavioral elements of a complex intervention from other elements, focusing on the action necessary to effect change [18]. It defines 4 social mechanisms that come together to enact the processes necessary for complex intervention: coherence, cognitive participation, collective action, and reflexive monitoring. Those integrating change must first make sense of the expectations and goals of the new practice (coherence) and then actively engage with it to appreciate its role and scope (cognitive participation). Where there is a decision to engage in change, critical to the ultimate success of its integration into standard practice, users must interact meaningfully with each other and relevant stakeholders to enable its use (collective action), followed by an evaluative phase that feeds back to iteratively inform practice (reflexive monitoring) [19].

A recent systematic literature review conducted by May et al [20] highlighted that although NPT had been used on occasion to investigate formal changes in professional roles, few have explored NPT constructs from a macro perspective. They suggested that this might be a valid future area of consideration. Although each of the domains of NPT may be necessary for ultimate success, health professional associations have a distinct role, from a macro perspective, in both coherence and cognitive participation, essentially the sensemaking and relational work required to enact change. Considering NPT at this level may strengthen the theory and suggest unique factors to be considered when looking beyond individuals within a team.

### Case Study Approach

Case study research values a comprehensive and multifaceted exploration of an uncontrolled and yet formally defined area, rather than a constrained and variable-driven correlational investigation [21]. Thus, it tends to value depth over breadth in the substance and applicability of its results. In consideration of how health professional associations address disruptive technology, the use of a case study approach was indicated based on the following factors: (1) a bounded case and relevant context are readily defined, (2) context is essential to the understanding of the phenomenon, (3) relevant insight cannot likely be gleaned from one source, and (4) a theory exists to provide a worthwhile foundation on which to build. A well-selected case or set of cases can provide nuanced context-dependent insight that might not be readily available when focusing on the generalizability of a more statistical approach, when “the boundaries between phenomenon and context may not be clearly evident” [21-23].

### Defining the Cases

#### Case Selection

A collective of 4 instrumental cases within 2 related areas of health care practice was sought. The intention was to identify fields that are traditionally heavily reliant on technology and where AI is currently being explored. Engaging multiple health professional associations with a stake in interdependent practice environments also highlights the interprofessional dynamic that could inform change integration.

**Figure 1:**
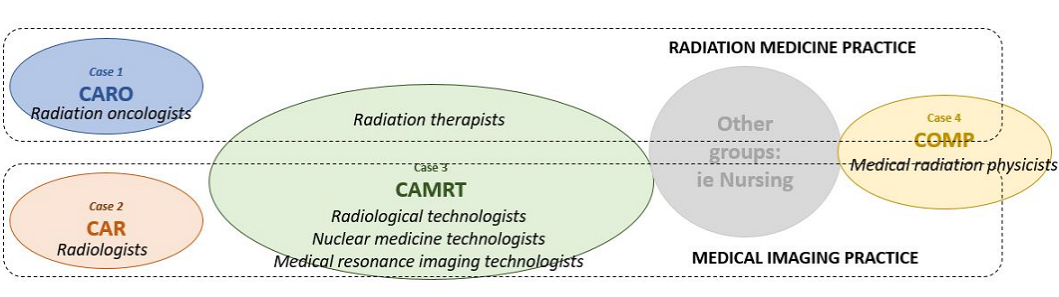
Relational case and context. CAMRT: Canadian Association of Medical Radiation Technologists; CAR: Canadian Association of Radiologists; CARO: Canadian Association of Radiation Oncology; COMP: Canadian Organization of Medical Physicists.

The related fields of medical imaging and radiation medicine were chosen as the overlapping case contexts, with 4 involved health professional associations selected as cases: the Canadian Association of Radiation Oncology (CARO), the Canadian Association of Radiologists (CAR), the Canadian Organization of Medical Physicists (COMP), and the Canadian Association of Medical Radiation Technologists (CAMRT). The contextual relationship of these health professional associations and their members within their respective frontline practice environments are represented graphically in Figure 1.

#### Radiation Medicine

Radiation therapy is one of the main pillars of cancer care, in addition to surgery and chemotherapy. It involves focused delivery of ionizing radiation, which is carefully targeted to a defined anatomical area, capitalizing on the ability of radiation to selectively kill actively reproducing cells, namely, those that are cancerous, while attempting to minimize side effects to normal healthy tissues. Oncologists diagnose the cancer and prescribe the radiation treatment, whereas therapists map the plan for treatment and deliver it, commonly over the course of several weeks. Physicists focus on how radiation behaves in the body and ensure calibrated functioning of related machines and treatment planning software.

There are 47 radiation treatment facilities in Canada, which are operated by provincial governments in the country’s publicly funded health care system [24]. Facilities range from large academically affiliated departments in dedicated multidisciplinary cancer centers to smaller hubs within community hospitals and some individual satellite treatment bunkers. In 2014, approximately 118,350 courses of radiation therapy treatment were administered in Canada, and it is estimated that 50% of all cases of cancer could likely benefit from radiation therapy at some point in the treatment trajectory [25].

#### Medical Imaging

Medical imaging is the process of creating visual representations of the inside of the human body (primarily using radiation but also involving other modalities such as ultrasound and magnetic resonance) to identify and characterize a disease or injury (diagnostic radiology). The related area of interventional radiology can also involve minimally invasive imaging techniques to assist in therapeutic care. Technologists position patients and acquire the imaging studies, whereas radiologists interpret the images, often for the purposes of diagnosis, and consult with referring physicians [26]. Similar to radiation medicine, physicists commission and maintain image acquisition and visualization infrastructure.

In 2012, over 6 million medical resonance and computed tomography examinations were performed in Canada, almost twice the number as the previous decade [26]. Although many imaging examinations are performed in large medical imaging departments in publicly funded hospitals and community clinics, approximately 10% of imaging facilities are small, private, stand-alone businesses [27].

Technology has always been, and remains, at the heart of contemporary radiation medicine and medical imaging practice [28,29]. The professions at the heart of these fields are adept at adapting to novel technologies, being heavily reliant on both computer software systems and large and complex medical machines to provide care. Examples of automation and machine learning in radiation medicine and medical imaging have emerged in recent years, and providers are becoming familiar with the ways in which AI could augment care and necessitate a shift in professional roles [15,16,28-33].

Although there has been significant research and development work regarding the potential for AI strategies [5,34-37] to facilitate more efficient and higher quality care, little attention has been paid to the impact on the professionals who engage in care delivery.

#### Cases and Replication Logic

Table 1 provides a case logic table that summarizes the relevant comparative characteristics between the 4 health professional associations, highlighting similarities and differences that can inform the use of data to make theoretical and practical inferences [21].

The characteristics of the 4 cases suggest that CARO and CAR reflect similarly positioned and sized physician bodies, suggesting similar professional autonomy and influence. The fact that CAR employs a robust administrative structure, however, suggests that there may exist different bandwidths between CAR and CARO to formally support coordinated professional initiatives. CAMRT and COMP differ from CAR and CARO and from each other in the size of their relevant membership and associated professional backgrounds but align in the intrahealth professional association heterogeneity of their membership, with representation in both medical imaging and radiation medicine. For CAMRT, as a significantly larger allied health group than the other groups, although with traditionally less autonomy in defining practice in a hierarchical health care environment, it may perceive itself as having less agency in defining its own future practice, looking to the other organizations for guidance in how to position themselves within the AI landscape. COMP differs in its current lack of regulation but is similar to the CAMRT in the different subspecialties under its purview, with members practicing in both medical imaging and radiation medicine. COMP also represents a professional group that tends to hold a large stake in the commercialization efforts around novel technologies. This may suggest a more robust baseline understanding of the nature of AI and its impact across the organization, reinforcing a theoretical replication in terms of the maturity of a perspective on AI manifested across COMP initiatives and membership.

**Table 1:** Case logic table.

Elements of organization		CAMRT^a^	CARO^b^	CAR^c^	COMP^d^
Year founded		1942	1988	1937	1989
**Membership**					
	Professions	Radiation therapy (also diagnostic imaging professions)	Radiation oncology physicians (also associate membership offered to other related professions—radiation therapy, medical physics)	Physicists or engineers in medicine	—^e^
	Number of members	~12,000 (~15% radiation therapist)	~500	~300	~300 (~50% radiation physics)
	Mandatory?	No	No	No	No
Regulation of practice?		No; provincially, where required	No; provincially, required	No; provincially, required	No, not regulated (in progress in most provinces)
Entry-to-practice oversight?		Yes, sets competency profiles and certification examinations	No (managed by RCPSC^f^); oversees liaison specialty committee to inform entry-to-practice	No (managed by RCPSC); oversees liaison specialty committee to inform entry-to-practice	No (managed by CCPM^g^—common management and MOU^h^)
Entry-to-practice requirements		Primarily undergraduate	MD^i^; medical residency	MD; medical residency	Primarily PhD; primarily residency
**Organizational structure**					
	By-laws	Yes	Yes	Yes	Yes
	Board of directors	Yes, elected volunteers; practicing professional members	Yes, elected volunteers; practicing professional members	Yes, elected volunteers; practicing professional members	Yes, elected volunteers; practicing professional members
	Administration	CEO^j^, directors, staff of 20	Secretariat central (association management firm)	—	Executive director, staff of 3 (joint with CCPM)
	Provincial-level counterparts?	Yes, provincial member associations (sometimes joint body with provincial regulatory arm)	No	Yes, in some provinces	No
**Professional guidance**					
	Code of ethics	Yes	Yes	No	Yes
	Practice guidelines	Yes	Yes	Yes	Yes
	Education committee	Yes	Yes	Yes	Yes
	Formal continuing education portfolio	Yes	No	Yes	No
	Professional practice or affairs committee	Yes	Yes	Yes	Yes
	Affiliated academic journal	Yes*; Journal of Medical Imaging & Radiation Sciences*	No (collaborates with European Society in *Radiation Oncology)*	—	No

^a^CAMRT: Canadian Association of Medical Radiation Technologists.

^b^CARO: Canadian Association of Radiation Oncology.

^c^CAR: Canadian Association of Radiologists.

^d^COMP: Canadian Organization of Medical Physicists.

^e^Not available.

^f^RCPSC: Royal College of Physicians and Surgeons of Canada.

^g^CCPM: Canadian College of Physicists in Medicine.

^h^MOU: Memorandum of understanding.

^i^MD: Doctor of Medicine.

^j^CEO: Chief executive officer.

#### Bounds to the Cases

**Figure 2:**
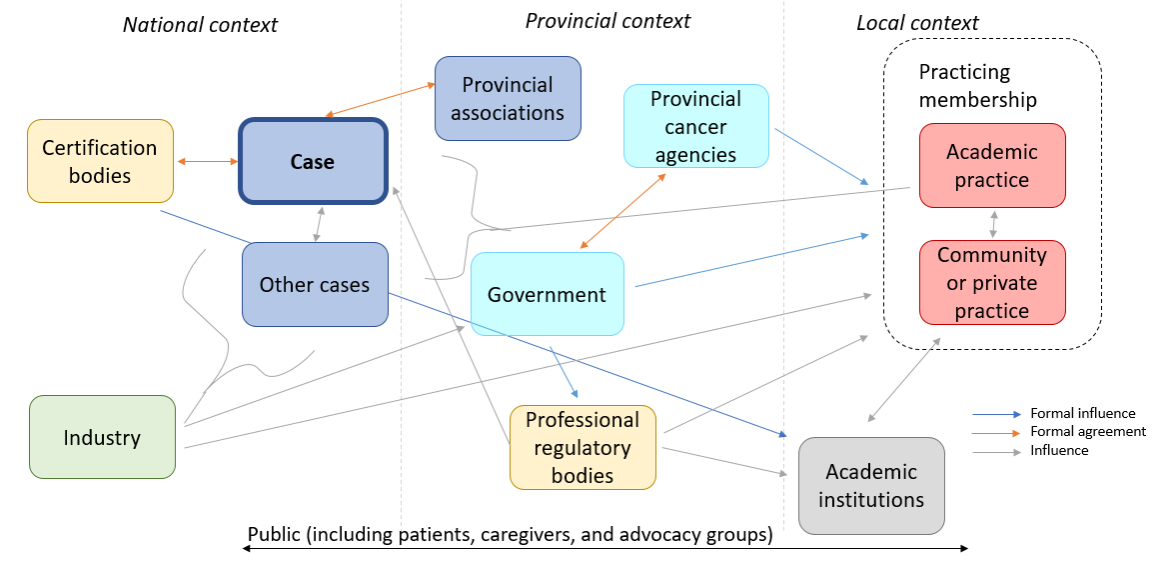
Artificial intelligence strategy integration in Canadian radiation therapy and medical imaging fields.

To bound the cases spatially and temporally, the following parameters will be defined. Temporally, the timeline of this work will be bounded by the individual health professional association’s current, immediately previous, and pending strategic plans. Although this may differ slightly for each health professional association, the timeline will run from approximately 2012 to the present time for each health professional association (including documentation prepared for future strategic plans that has been prepared by the time of data collection). With respect to bounds placed on scope, or spatial bounds, consideration will be primarily on the health professional associations’ consideration of AI-enabled practice. Other stakeholders, such as those depicted in the preliminary network map in Figure 2, will be captured through insights into how the health professional association engages them and the nature of that engagement.

### Data Collection

For each case, 3 phases of data collection will be pursued: document review (phase 1), interviews (phase 2), and observation (phase 3), with a cross-cutting reflexivity phase that will span study design, data collection, and analysis.

#### Phase 1: Document Review

Phase 1 will consist of a review of formal health professional association documentation, that which is available publicly through health professional association websites and that which will be sought through the chief executive officer or other administrative contact identified as the representative of the organization at the time of agreeing to participate in the study and able to facilitate documentation identification and access. A preliminary list of documentation sought is provided in Textbox 1. Additional documents may be identified by key informants at each health professional association, namely, those engaged in phase 2 (interviews). The final compiled list will be reviewed with the administrative lead (ie, chief executive officer) before concluding data collection to ensure that all relevant documents have been captured.

Documents will be uploaded to a case study database managed using Excel. The database will be organized by data type, medium, and year and potentially subcategorized in other ways depending on the nature and volume of collected documentation [21].

**Preliminary list of documentation and content sought in document analysis.**

**Documentation**

- Strategic plans (previous, current, and upcoming)
- Terms of reference for relevant task forces and working groups
- Meeting minutes from strategic and artificial intelligence (AI)–related meetings (board meetings, task group meetings)
- Professional competency profiles maintained or administered by PA (including Royal College of Physicians and Surgeons in Canada documentation maintained by Canadian Association of Radiologists and Canadian Association of Radiation Oncology)
- Annual reports (starting at the implementation of previous Strategic Plan [2012])
- Programmatic review (continuing professional development offerings, summits, scientific meeting themes etc)
- Position statements, white papers, and other formal communications regarding AI published or released by PA
- Original posts by health professional associations Twitter accounts since 2012

**Content Sought in Document Review**

- Level, nature, or timing of attention given to AI within the PA
- Degree to which AI is considered in entry-to-practice training or other educational offerings
- Formal collaborations with (or even reference to or consideration of) other PAs or stakeholders
- Volume, nature, or scope of formal opportunities presented to membership or to external stakeholders, by the PA, to communicate a position on AI

#### Phase 2: Interviews

Between 3 and 5 purposively selected, semistructured interviews will be conducted for each case [21]. An interview guide will be used, with potential probes included to help direct the conversation, as necessary (refer to Multimedia Appendix 1 for the interview question script). Interviews, scheduled for 60 minutes, will be conducted by telephone or in-person by the lead investigator (CG) and audio-recorded for subsequent transcription. Written consent will be sought and reconfirmed verbally at the time of the interview. Notes will be taken as needed. The interview guide will be piloted with at least one leader in another health professional association not participating in this study.

The full interview guide, minus probes, will be provided via email to each participant at least 48 hours before the interview. The board president of each health professional association will be the first to be invited to participate in an interview for each case, followed by the administrative lead. Both of these interviews will involve discussion of other relevant interviews that might follow (ie, snowball sampling), including the head of any relevant technology-focused task force or committee and any identified key subject matter consultants within the health professional association structure or formally engaged on behalf of the health professional association.

Interview audio-recordings, transcripts, and notes will be maintained and indexed in the case study database.

#### Phase 3: Observation

A third phase of data collection will involve the observation of strategically identified health professional association meetings that relate to its strategic stance on AI and approach to addressing it with members and stakeholders. The spectrum of the health professional association board, board subcommittee, health professional association committee, and task force meetings will be reviewed, and the following will be identified within a 12-month period:

1. Organizational strategic meetings (board meetings and strategic planning retreats)
2. Focused technology or AI-related strategic or operational meetings (task force meetings and AI-themed conference planning meeting).

A 12-month window will represent a normal cycle of standing meetings for a health professional association, even those held only annually. Committee chairs and/or administrative assistants will be approached to facilitate access to relevant meetings.

Observation will consist of the lead investigator (CG) attending the scheduled meeting by teleconference as a nonparticipant observer, with the written consent and permission of the meeting chair and the awareness of all participating members. Field notes will be taken to maintain a record of any discussions, debates, and/or action items relating to AI and will be maintained in the case study database.

### Reflexivity

Throughout the process of study design, data collection, and analysis, the lead investigator (CG) will engage in reflective exercises, including journaling, to consider how her position within the case contexts might impact the data and how the data are interpreted. As a professional member and past board member of one of the health professional associations (CAMRT), any increased identification with this group or acknowledgment of traditional professional hierarchies, culture, or knowledge base in any study interviews might warrant consideration with the broader study investigators and in reporting on the study.

### Data Analysis

A hybrid inductive and deductive model that uses NPT as an analytical framework will be used to analyze the data. This avoids the need to force data that do not naturally fit with theory and will be of value in informing the macro applications of the NPT model. The inductive element will be managed using NVivo software (QSR International) and will involve an inductively coded and concept-driven thematic analysis [38-40], first within cases and subsequently between cases using a constant comparison method. Concurrent to the thematic analysis, a network map will be prepared that reflects how information and knowledge flows between identified stakeholders, using arrows and notes to suggest linkages and the nature of interactions (preliminary network map included as Figure 2) [41]. Document data can then be integrated within those themes to further inform the themes and demonstrate where action aligns with perception or intent. Documentation that provides overarching perspectives or positions on AI, such as position papers or strategic plans, will be prioritized to provide initial scaffolding to guide analysis. Observation data will primarily serve to highlight the nature of any internal information sharing, consensus building, debate, or other collaborative work that might have contributed to the actions or direction taken by the health professional association as it relates to AI.

Using the framework of the domains and individual constructs of the NPT, fit will be assessed between the theoretical framework and the data as interpreted through pattern matching [21]. Emergent themes will be mapped deductively against the domains of NPT to assess fit and identify areas of discordance. The network map will be annotated according to knowledge acquisition or sharing efforts relating to different constructs. Areas where coherence or cognitive participation efforts align or differ between cases will be considered, and the fit and tensions between study themes and NPT will also be considered.

The study will be reported using the guidelines suggested for organizational case studies, as defined by Rodgers et al [42] and accessible via the EQUATOR (Enhancing the Quality and Transparency of Health Research) Network (Multimedia Appendix 2).

## Results

As of January 2021, we have conducted the majority of the interviews expected to be required for this study (n=17), with representation across the 4 health professional associations. Of these 17 interviews, 15 (88%) have been transcribed. The document review is underway, with 2 of the 4 health professional association websites fully reviewed for related documents, and 2 health professional associations have contributed additional documents. Twitter feeds for all health professional associations have also been reviewed, dating back to the inception of each health professional association’s account, but will require updating for future content within the data collection period. Observation opportunities have been challenged by competing priorities during the COVID-19 pandemic and may require revisiting.

## Discussion

The research will be reported as a linear analytic process, prioritizing the cross-case analysis to highlight both the contributions to theory and strategies and their rationale for those health professional associations approaching change themselves [21]. The research findings will be presented according to the NPT constructs in the coherence and cognitive participation domains, considering the internal and collaborative efforts of a health professional association to define its perspective and role. The collective action and reflexive monitoring domains of NPT will be the primary lens for considerations of future practice models, competencies, and potential changes to the scopes of practice and education for which health professional associations are responsible.

Following this presentation of data, implications for NPT will be presented, suggesting where this study might reinforce its constructs more strongly at the often-neglected macro level of the health professional association or introduce tensions that might require further attention. Implications for practice, limitations of the case study approach, and potential for future work will be highlighted.

It is anticipated that health professional associations will be at varying stages of considering AI and its impact on the profession and its practice, which may challenge direct comparison of approach or alignment with NPT. Factors impacting this may include the perceived impact on the profession; the perceived contribution of professional expertise to informing AI in the relevant practice environment; and other organizational and professional characteristics such as size, administrative structure, and competing professional priorities. The use of mechanisms such as task forces, position papers, and continuing education forums (webinars and conference panels) will likely vary depending on the expertise within the health professional association and the priority assigned to AI. Although academic work done within the professions might be of value in building a sense of the future impact of AI, and engagement with industry may also contribute to perspectives, it is anticipated that formal collaborative consideration between health professional associations of how the practice environment might change will be minimal and superficial. Thus, the collaborative constructs within NPT may be impacted by a passive lack of engagement between professions, as opposed to an active resistance to considering the scopes of practice. Given the nebulous nature of AI strategies (in terms of their origin and application) and the current state of implementation in practice, it is likely that insight into later stages of NPT, related to action and monitoring, will be based primarily on anticipated future action rather than reflection on past or ongoing events.

The fields of radiation medicine and medical imaging are 2 areas—related in many ways but unique in others—that rely heavily on technology and are already exploring the potential of AI. A picture of how such professions envision themselves as stakeholders in approaching disruptive technology can help to direct future initiatives to prepare for evolution and can potentially inform relevant policy and practice change as AI strategies are introduced. How different stakeholders, including other health professional associations whose professions interact in the same practice environment, are engaged in informing sensemaking and preparation for change can also suggest what influences contribute to professional perspectives at the front lines of health care practice and, ultimately, to the nature of AI-enabled practice.

As AI is increasingly positioned to significantly disrupt many aspects of the society, it is incumbent on those whose roles may be replaced, displaced, augmented, or otherwise impacted by AI to be proactive in preparing for associated change. Using NPT as a framework to consider the behavioral aspects of change at the level of the profession, rather than the individual, is important in establishing professional readiness and adaptation for AI.

## Appendix

Multimedia Appendix 1 Interview script.

Multimedia Appendix 2 Consensus standards for the reporting of organizational case studies.

## Abbreviations

AI: artificial intelligence
CAMRT: Canadian Association of Medical Radiation Technologists
CAR: Canadian Association of Radiologists
CARO: Canadian Association of Radiation Oncology
COMP: Canadian Organization of Medical Physicists
EQUATOR: Enhancing the Quality and Transparency of Health Research
NPT: normalization process theory

## References

[1] (2017). Challenge Ahead: integrating robotics, artificial intelligence, and 3D printing technologies into Canada's healthcare systems. Standing Senate Committee on Social Affairs, Science and Technology.

[2] Russell S (2009). Artificial Intelligence: A Modern Approach.

[3] Copeland BJ Artificial intelligence.

[4] Mehta N, Devarakonda MV (2018). Machine learning, natural language programming, and electronic health records: the next step in the artificial intelligence journey?. J Allergy Clin Immunol.

[5] Esteva A, Kuprel B, Novoa RA, Ko J, Swetter SM, Blau HM, Thrun S (2017). Dermatologist-level classification of skin cancer with deep neural networks. Nature.

[6] Hosny A, Parmar C, Quackenbush J, Schwartz LH, Aerts HJ (2018). Artificial intelligence in radiology. Nat Rev Cancer.

[7] Vyas M, Thakur S, Riyaz B, Bansal K, Tomar B, Mishra V (2018). Artificial Intelligence: the beginning of a new era in pharmacy profession. Asian J Pharm.

[8] Hughes W, Hughes C (2013). Professionalism and professional institutions in times of change. Build Res Inf.

[9] Evetts J (2016). The Sociological Analysis of Professionalism. Int Socio.

[10] Greenwood R, Hinings CR, Suddaby R (2002). Theorizing change: the role of professional associations in the transformation of institutionalized fields. Acad Manage J.

[11] Glauser W (2017). Artificial intelligence, automation and the future of nursing. Can Nurse.

[12] Jha S, Topol EJ (2016). Adapting to artificial intelligence: radiologists and pathologists as information specialists. J Am Med Assoc.

[13] Booth R (2016). Informatics and nursing in a post-nursing informatics world: future directions for nurses in an automated, artificially intelligent, social-networked healthcare environment. Nurs Leadersh (Tor Ont).

[14] Patel VL, Shortliffe EH, Stefanelli M, Szolovits P, Berthold MR, Bellazzi R, Abu-Hanna A (2009). The coming of age of artificial intelligence in medicine. Artif Intell Med.

[15] Tang A, Tam R, Cadrin-Chênevert A, Guest W, Chong J, Barfett J, Chepelev L, Cairns R, Mitchell JR, Cicero MD, Poudrette MG, Jaremko JL, Reinhold C, Gallix B, Gray B, Geis R, Canadian Association of Radiologists (CAR) Artificial Intelligence Working Group (2018). Canadian association of radiologists white paper on artificial intelligence in radiology. Can Assoc Radiol J.

[16] Thompson RF, Valdes G, Fuller CD, Carpenter CM, Morin O, Aneja S, Lindsay WD, Aerts HJ, Agrimson B, Deville C, Rosenthal SA, Yu JB, Thomas CR (2018). Artificial intelligence in radiation oncology: a specialty-wide disruptive transformation?. Radiother Oncol.

[17] (2019). Developing and evaluating complex interventions. Medical Research Council and National Institute for Health Research.

[18] Johnson MJ, May CR (2015). Promoting professional behaviour change in healthcare: what interventions work, and why? A theory-led overview of systematic reviews. BMJ Open.

[19] May C, Rapley T, Mair F, Treweek S, Murray E, Ballini L (2015). Normalization process theory on-line users’ manual, toolkit and NoMAD instrument. Normalization Process Theory.

[20] May CR, Cummings A, Girling M, Bracher M, Mair FS, May CM, Murray E, Myall M, Rapley T, Finch T (2018). Using Normalization Process Theory in feasibility studies and process evaluations of complex healthcare interventions: a systematic review. Implement Sci.

[21] Yin RK (2017). Case Study Research and Applications: Design and Methods.

[22] Platt J (2016). “Case Study” in American methodological thought. Curr Socio.

[23] Flyvbjerg B (2016). Five misunderstandings about case-study research. Qual Inq.

[24] Agnew A, Galal M (2016). Implementing the Canadian Partnership for Quality Radiotherapy (CPQR) guidelines in a radiotherapy department. Physica Medica.

[25] Delaney G, Jacob S, Featherstone C, Barton M (2005). The role of radiotherapy in cancer treatment: estimating optimal utilization from a review of evidence-based clinical guidelines. Cancer.

[26] Canadian Institute for Health Information (2007). Medical imaging in Canada.

[27] (2018). The Canadian medical imaging inventory, 2017. Canadian Agency for Drugs and Technologies in Health.

[28] Ogura T, Sato M, Ishida Y, Hayashi N, Doi K (2014). Development of a novel method for manipulation of angiographic images by use of a motion sensor in operating rooms. Radiol Phys Technol.

[29] Jaffray DA (2012). Image-guided radiotherapy: from current concept to future perspectives. Nat Rev Clin Oncol.

[30] Kane G (2007). Step-by-step: a model for practice-based learning. J Contin Educ Health Prof.

[31] White E, Kane G (2007). Radiation medicine practice in the image-guided radiation therapy era: new roles and new opportunities. Semin Radiat Oncol.

[32] Gillan C, Milne E, Harnett N, Purdie TG, Jaffray DA, Hodges B (2018). Professional implications of introducing artificial intelligence in healthcare: an evaluation using radiation medicine as a testing ground. J Radiother Pract.

[33] Gillan C, Wiljer D, Harnett N, Briggs K, Catton P (2010). Changing stress while stressing change: the role of interprofessional education in mediating stress in the introduction of a transformative technology. J Interprof Care.

[34] Goto T, Camargo CA, Faridi MK, Freishtat RJ, Hasegawa K (2019). Machine learning-based prediction of clinical outcomes for children during emergency department triage. JAMA Netw Open.

[35] Janke AT, Overbeek DL, Kocher KE, Levy PD (2016). Exploring the potential of predictive analytics and big data in emergency care. Ann Emerg Med.

[36] Jilani T, Housley G, Figueredo G, Tang P, Hatton J, Shaw D (2019). Short and long term predictions of hospital emergency department attendances. Int J Med Inform.

[37] Mullaney T (2017). How IBM Watson could help identify patients for homecare. Home Health Care News.

[38] Charmaz K, Belgrave L, Gubrium JF, Holstein JA, McKinney KD (2012). Qualitative interviewing grounded theory analysis. Handbook of Interview Research: The Complexity of the Craft (2nd Ed).

[39] Clarke V, Braun V, Hayfield N, Smith JA (2015). Thematic analysis. Qualitative Psychology: A Practical Guide to Research Methods.

[40] Brinkmann S, Kvale S (2015). InterViews: Learning the Craft of Qualitiative Research Interviewing.

[41] Mitchell RK, Agle BR, Wood DJ (1997). Toward a theory of stakeholder identification and salience: defining the principle of who and what really counts. Acad Manage Rev.

[42] Rodgers M, Thomas S, Harden M, Parker G, Street A, Eastwood A (2016). Developing a methodological framework for organisational case studies: a rapid review and consensus development process. Health Serv Deliv Res.

