# Supplementary material for: Health Care Professional Association Agency in Preparing for Artificial Intelligence: Protocol for a Multi-Case Study
Source: JMIR Res Protoc. 2021 May 19;10(5):e27340. doi: 10.2196/27340 (PMC8173392; doi:10.2196/27340)
Supplement: Multimedia Appendix 1 [file resprot_v10i5e27340_app1.docx]

Appendix I: Interview Question Script

1. To start off, can you give me an idea of your role within [HPA], and the background of your engagement with the organization?
2. What future role do you believe [HPA] anticipates will be played by AI broadly in healthcare and within [medical imaging or radiation therapy] care?
   - What are the perceived associated benefits and challenges to healthcare?
   - How was this perception conceived?
3. What do you believe [HPA] perceives to be the impact of AI on the [radiation oncology / radiology / medical physicist / medical radiation technology] professions?
   - In terms of front-line practice
   - In terms of relations to other groups
   - In terms of changes to professional scope/preparation?
4. Do you believe there has been a concerted effort on behalf of [HPA] to instill a certain mindset in its [radiation oncology / radiology / medical physicist / medical radiation technology] membership with respect to AI?
   - If so, what is this mindset and how was it conceived?
   - What do you believe the prevalent mindsets to be at present? Have they evolved over time? Do you envision them changing in the future?
   - Do you believe [HPA] perceives of AI as a threat to the profession and its practice boundaries?
5. How do you believe [HPA] perceives its role in preparing its membership for AI?
   - What priority is assigned within [HPA] to realizing this role?
6. What do you see as being the process taken by [HPA] in preparing for AI?

- Where is the profession in the process at this point in time?
- What mechanisms or processes does [HPA] employ, if any, to keep a finger on the pulse of the status of this change?
  - With respect to advancements in AI?
  - With respect to integration in the practice environment?
- degree/nature of consideration/collaboration with other stakeholders and professions (PAs, academia, industry, healthcare organizations) in defining and realizing future practice (opportunities, challenges, and tensions)
- Describe the nature of any stakeholder relationships – informal or formal? One-way information gathering, information sharing, or collaborative partnerships or associated ventures?
