# Supplementary material for: Health Care Professional Association Agency in Preparing for Artificial Intelligence: Protocol for a Multi-Case Study
Source: JMIR Res Protoc. 2021 May 19;10(5):e27340. doi: 10.2196/27340 (PMC8173392; doi:10.2196/27340)
Supplement: Multimedia Appendix 2 [file resprot_v10i5e27340_app2.docx]

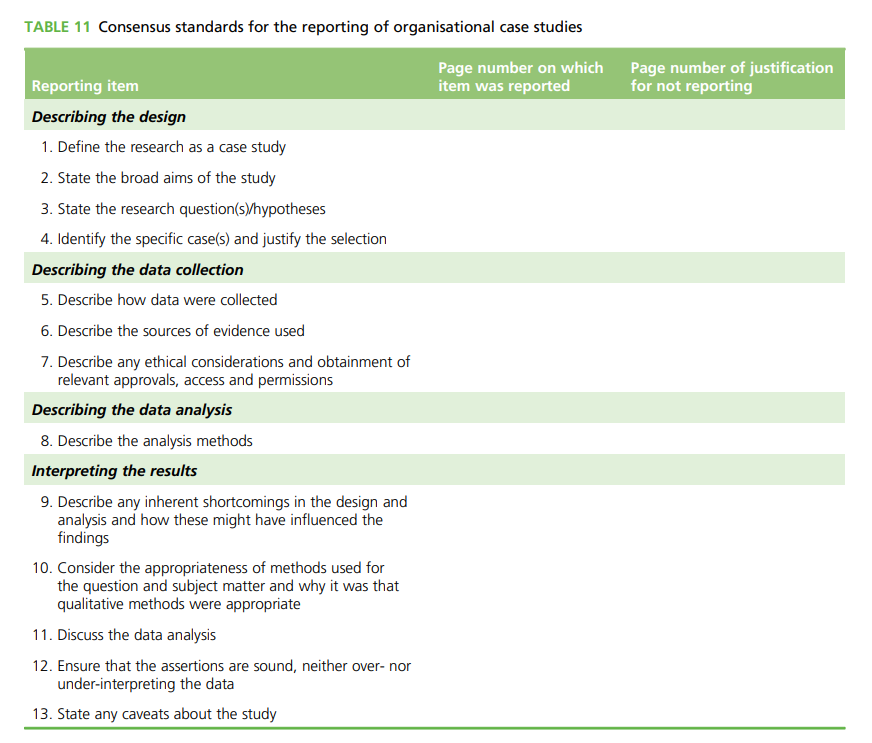


p8, p15, p18

As a protocol paper, these elements could only be addressed from an anticipatory or planning perspective – the ‘Discussion’ section reflects on some expectations of findings and limitations regarding applicability to theory etc (p19-21)

p18-19

p15-18

p15-18

p11-15

p8

p8

p7-8
